# Association of *BRCA1* promoter methylation with sporadic breast cancers: Evidence from 40 studies

**DOI:** 10.1038/srep17869

**Published:** 2015-12-08

**Authors:** Li Zhang, Xinghua Long

**Affiliations:** 1 Zhongnan Hospital of Wuhan University, Wuhan, 430071, China

## Abstract

Breast cancer susceptibility gene 1 (*BRCA1*) located at chromosome 17q12-21 is a classic tumor suppressor gene, and has been considered as a significant role in hereditary breast cancers. Moreover, numerous studies demonstrated the methylation status of CpG islands in the promoter regions of *BRCA1* gene was aberrant in patients with sporadic breast tumors compared with healthy females or patients with benign diseases. However, these conclusions were not always consistent. Hence, a meta-analysis was performed to get a more precise estimate for these associations. Crude odds ratio with 95% confidence interval were used to assess the association of *BRCA1* promoter methylation and the risk or clinicopathologic characteristics of breast cancers under fixed or random effect model. A total of 40 studies were eligible for this present study. We observed the frequency of BRCA1 promoter methylation was statistically significant higher in breast cancers than non-cancer controls. Furthermore, BRCA1 methylation was statistically associated with lymph node metastasis, histological grade 3, ER(-), PR(-), triple-negative phenotype, and decreased or lack levels of BRCA1 protein expression. In conclusion, this study indicated that BRCA1 promoter methylation appeared to be a useful predictive or prognostic biomarker for breast cancers in clinical assessment.

Breast cancer is one of the common malignant tumors for females worldwide. As we know that breast carcinomas belong to a heterogeneous group of tumors, not only for multiple biological behaviors, but also for various therapeutic responses and clinical outcomes. Though the exact pathogenic mechanism is not fully understood as yet, researchers have been demonstrating that multiple factors are involved in the initiation and progression of breast tumors, such as germline mutation and epigenetic alteration. DNA promoter methylation, as one of main ways of epigenetic alteration, has been investigated for breast cancer detection, prognosis and treatment^1^. There is mounting evidence that methylation status of CpG islands in cancer-related genes promoters, especially tumor suppressor genes, is distinct in breast cancer patients compared with healthy women or patients with benign breast disease^2^,^3^,^4^. For instance, the *BRCA1* promoter was prone to methylate in peripheral blood DNA of sporadic breast cancer patients compared with unaffected controls^5^.

Breast cancer susceptibility gene 1 (*BRCA1*) located at chromosome 17q12-21 is a classic tumor suppressor gene^6^, and plays a crucial role in the processes of DNA repair, homologous recombination, checkpoint control of cell cycle and transcription^7^. Germline mutations of *BRCA1* account for 30–40% hereditary breast carcinomas^8^. However, somatic mutations of *BRCA1* are rare in sporadic cases of breast cancer^9^,^10^,^11^. In addition, there are some evidences that lack or low expression of *BRCA1* protein is involved in the development of sporadic breast tumors^12^,^13^. Furthermore, it’s also noteworthy that several studies have shown a significant association between reduced expression of *BRCA1* protein and aberrant methylation status of CpG islands in the *BRCA1* promoter^6^,^12^,^13^,^14^, which indicates that promoter methylation may lead to transcriptional inactivation of *BRCA1* gene and contribute to breast carcinogenesis.

Over past few years, the prevalence of the hypermethylated *BRCA1* promoter in sporadic breast cancers has been reported to fall in the range from 5.2% to 65.2%^13^,^15^. Iwamoto *et al.* showed that *BRCA1* promoter methylation in peripheral blood cells was correlated with elevated risk of *BRCA1*-methylated breast cancers^16^. Interestingly, the methylation frequencies of *BRCA1* promoter were different in breast cancer tissues, paired adjacent normal tissues and peripheral blood cells derived from breast cancers and unaffected women^17^. Furthermore, during the past decade, a considerable amount of studies have been going to elucidate the association between *BRCA1* promoter methylation and clinicopathological characteristics of breast cancer^18^,^19^. However, the research results are not always consistent. Therefore, we performed a meta-analysis to evaluate whether *BRCA1* gene promoter methylation is a risk factor for sporadic breast cancers, and elucidated the association of *BRCA1* promoter methylation with clinicopathological characteristics in patients with breast cancer.

## Materials and Methods

### Literature search strategy

We performed a comprehensive literature search from PubMed and EMBASE database (last search updated in August 2015) without language restrictions. The following search terms were used, (“*BRCA1*” or “Breast cancer susceptibility gene 1”) and (“methylation” or “DNA methylation” or “promoter methylation”) and (“breast cancer” or “breast carcinoma” or “breast tumor” “breast carcinogenesis”). In addition, we carried out a manual search for other relevant articles via the reference lists of eligible studies.

### Selection criteria

Eligible studies had to meet the following predefined criteria, (1) case-control studies evaluating the association between the prevalence of *BRCA1* promoter methylation and sporadic breast cancer risk; or clinical cohort studies evaluating the associations of *BRCA1* promoter methylation with clinicopathological features of sporadic breast cancer; (2) sufficient published data for calculating an odds ratio (OR) and 95% confidence interval (CI); (3) studies were confined to human female groups. It’s noteworthy that if the same study population was included in several different studies, we would only bring the most recent or comprehensive study into the meta-analysis.

### Data extraction

A standard protocol was applied to extract data. For every eligible study, the following data were extracted: the first author’s name, publication year, original country, methods for detecting methylation, the frequency of *BRCA1* promoter methylation in case and control groups, control characteristics, sample materials and so on.

### Statistical methods

The strength of the association between the *BRCA1* promoter methylation and sporadic breast cancer risk or clinicopathological features was assessed by OR with corresponding 95%CI. A chi-square-based Q test was applied to test heterogeneity among studies. The p value of the Q test was >0.1, which suggested a lack of statistically significant heterogeneity, and we used the fixed-effect model (Mantel-Haenszel method)^20^ to calculate pooled ORs. Otherwise, heterogeneity was present and the random-effect model (DerSimonian-Laird method)^21^ was more appropriate. Additionally, the degree of heterogeneity was also quantitatively assessed by *I*^*2*^-test, which the value of I^2^ ranged from 0 to 100% and was generally considered mild heterogeneity for *I*^2^ < 25%, moderate heterogeneity for 25%–50%, large heterogeneity for 50%–75%, and extreme heterogeneity for *I*^2^ > 75%^22^. Moreover, stratified-analyses were conducted based on ethnicity, methods for detecting methylation and sample materials to explore the potential source of heterogeneity. Sensitivity analyses, by which each study was omitted in each turn to confirm the influence of individual data set to the pooled OR, were implied to evaluate the robustness of the results. Furthermore, we estimated potential publication bias with funnel plot and Egger’s linear regression test. The funnel plot was visual symmetrical and the P-value of Egger’s test was greater than 0.05, which indicated that there were no statistically significant publication bias. All statistical tests in the meta-analysis were two-tailed and P-value 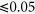 was considered statistically significant unless otherwise noted. Statistical analyses were performed with Review Manager 5.2 software recommended by Cochrane Collaboration and STATA software version 12.0.

## Results

### Study Characteristics

**Figure 1:**
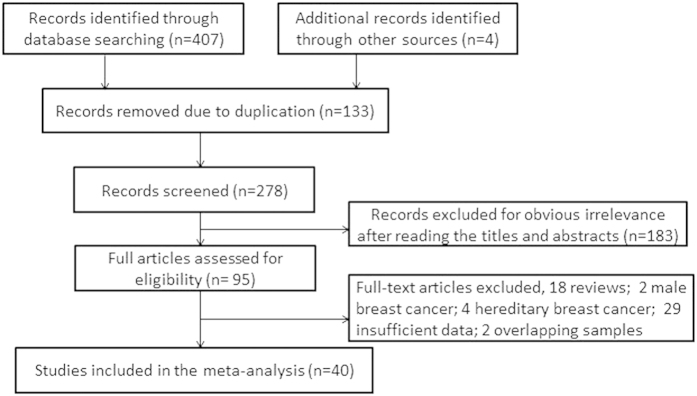
Flow diagram of study selection in this meta-analysis.

Based on the above selection criteria, 20 case-control studies , involving 2747 cases and 2256 controls^2^,^3^,^4^,^5^,^6^,^10^,^15^,^16^,^17^,^23^,^24^,^25^,^26^,^27^,^28^,^29^,^30^,^31^,^32^,^33^, were included to analyze the association between BRCA1 promoter methylation and sporadic breast cancer risk. Among these studies, 12 studies^4^,^6^,^10^,^15^,^17^,^23^,^24^,^25^,^26^,^27^,^28^,^32^ confirmed the status of *BRCA1* methylation in tissues derived from breast carcinoma, benign disease or normal breast epithelium. And the objects of 9 studies^2^,^3^,^5^,^16^,^17^,^29^,^30^,^31^,^33^ were the prevalence of *BRCA1* methylation in peripheral blood of breast cancer patients compared with cancer-free or healthy females. It’s worth noting that a study^17^ detected *BRCA1* methylation in tissues of tumor and normal breast epithelium, and peripheral blood from breast cancer patients and healthy women. Due to the different type of sample materials, we considered this study as two case-control studies. Furthermore, 30 clinical studies^3^,^4^,^6^,^12^,^13^,^14^,^16^,^18^,^27^,^29^,^30^,^31^,^32^,^33^,^34^,^35^,^36^,^37^,^38^,^39^,^40^,^41^,^42^,^43^,^44^,^45^,^46^,^47^,^48^,^49^ with a total of 5058 breast cancer patients met our selection criteria for analyzing the association between *BRCA1* promoter methylation and clinicopathological characteristics which included early age (<50 years) at diagnosis, premenopausal status, lymph node metastasis, histological grade 3, estrogen receptor (ER), progesterone receptor (PR), human epidermal receptor 2 (Her2), triple-negative phenotype and the expression of *BRCA1* protein. In short, our meta-analysis included 40 eligible articles, among which 20 articles were analyzed for the frequency of *BRCA1* promoter methylation in breast cancers compared with non-cancer controls, and 30 articles were analyzed for the association between *BRCA1* promoter methylation and clinicopathological features. What was noteworthy was that 10 articles^3^,^4^,^6^,^16^,^27^,^29^,^30^,^31^,^32^,^33^ not only studied the prevalence of *BRCA1* promoter methylation, but also elaborated clinicopathological characteristics in breast cancer patients with *BRCA1* promoter methylation versus *BRCA1*-unmethylated tumors. The flow diagram of study selection procedure was shown in Fig. 1. Every study characteristics were summarized in Tables 1 and 2.

### Meta-analysis results

#### Association between *BRCA1* promoter methylation and the risk of breast cancer

**Figure 2:**
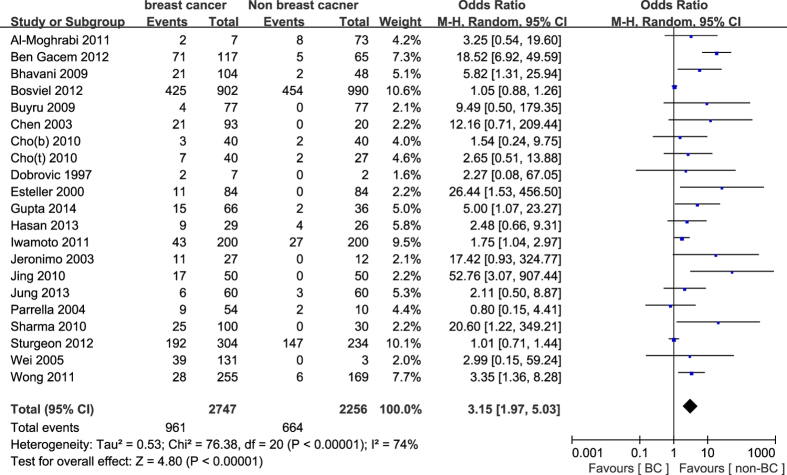
Forest plot for evaluating the association between BRCA1 promoter methylation and breast cancer risk. Random- effect model was used for the analysis.

In general, our study indicated that the frequency of *BRCA1* promoter methylation was statistically significant elevated in breast cancers compared with non-cancer controls (OR = 3.15, 95%CI 1.97–5.03, P < 0.001, Fig. 2). Because of large heterogeneity (P_H_ < 0.001, I^2^ = 74%), we explored the potential source of heterogeneity via stratified analysis based on sample materials, methods for detecting methylation and ethnicity. In the subgroup analysis about sample materials, the pooled OR for *BRCA1* methylation in breast cancer tissues compared with normal or benign tissues was 4.75 (95%CI 2.37–9.54, P < 0.001), and was higher than the pooled OR (OR = 1.87, 95%CI 1.19–2.96, P = 0.007) in peripheral blood of breast cancers compared with non-cancer controls. Furthermore, the frequency of *BRCA1* methylation by MSP method (OR = 6.79, 95%CI 3.05–15.11, P < 0.001) was significantly higher than other methods (OR = 1.53, 95%CI 1.09–2.14, P = 0.01). Meanwhile, the prevalence of *BRCA1* methylation in Asians (OR = 4.03, 95%CI 1.07–15.18, P = 0.04) was higher than that in Caucasians (OR = 3.16, 95%CI 1.78–5.62, P < 0.001) and in Australians (OR = 3.27, 95%CI 1.37–7.84, P = 0.008) in breast cancers compared with non-cancer controls. It’s worth mentioning that the degree of heterogeneity was apparently reduced in stratified analysis. The detailed results were summarized in Table 3.

#### Association of *BRCA1* promoter methylation with clinicopathological features of breast cancer

**Figure 3:**
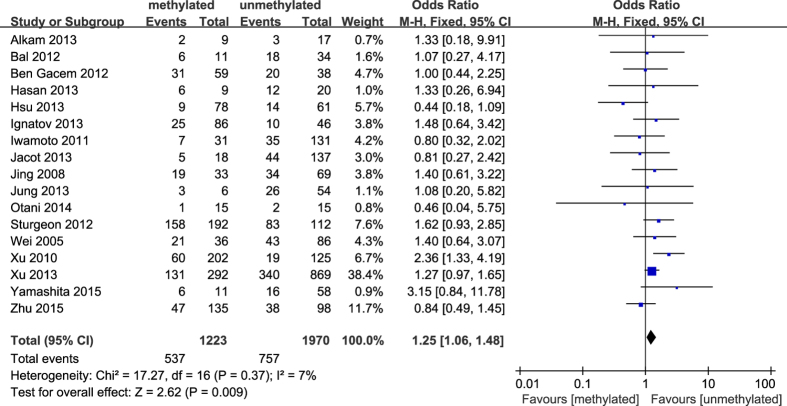
Forest plot for evaluating the association between BRCA1 promoter methylation and lymph node metastasis. Fix-effect model was used for the analysis.

**Figure 4:**
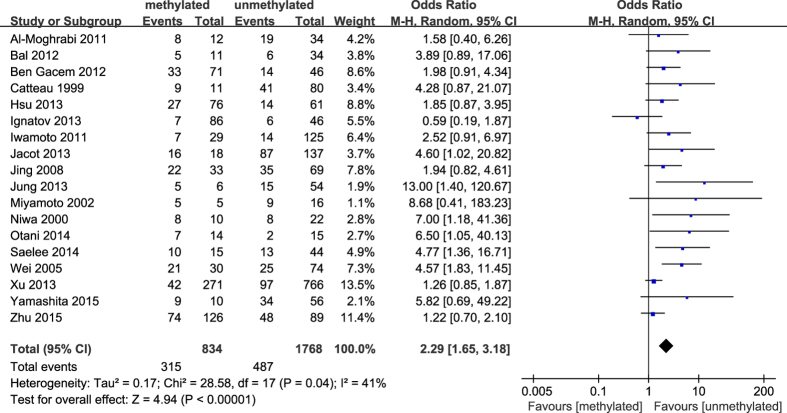
Forest plot for evaluating the association between BRCA1 promoter methylation and histological grade 3. Random-effect model was used for the analysis.

**Figure 5:**
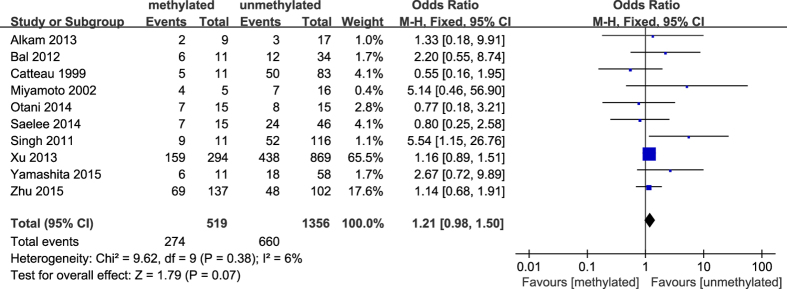
Forest plot for evaluating the association between BRCA1 promoter methylation and early age (<50 years) at diagnosis. Fix-effect model was used for the analysis.

**Figure 6:**
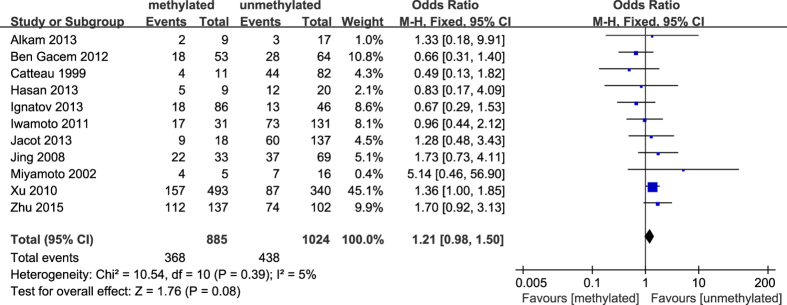
Forest plot for evaluating the association between BRCA1 promoter methylation and premenopausal status. Fix-effect model was used for the analysis.

**Figure 7:**
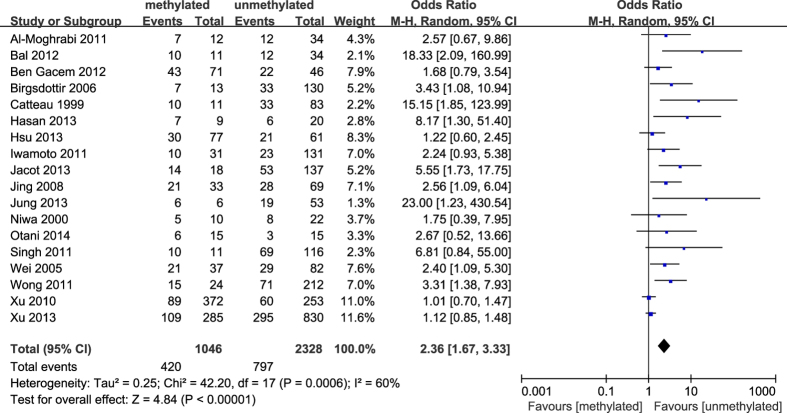
Forest plot for evaluating the association between BRCA1 promoter methylation and ER negative. Random-effect model was used for the analysis.

**Figure 8:**
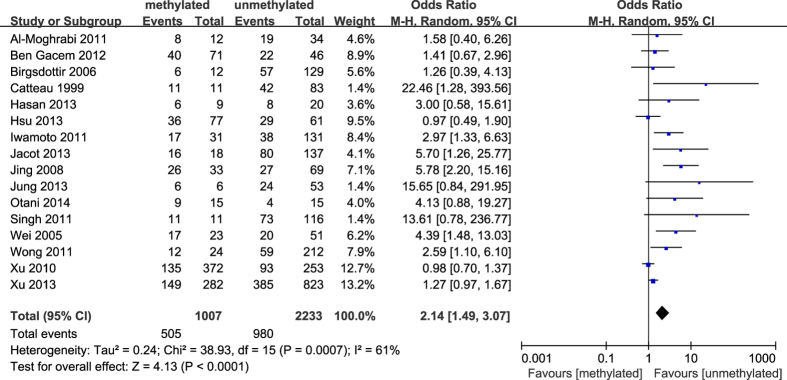
Forest plot for evaluating the association between BRCA1 promoter methylation and PR negative. Random-effect model was used for the analysis.

**Figure 9:**
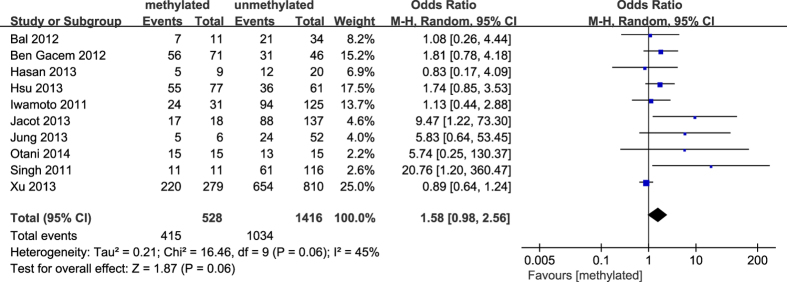
Forest plot for evaluating the association between BRCA1 promoter methylation and Her2 negative. Random-effect model was used for the analysis.

**Figure 10:**
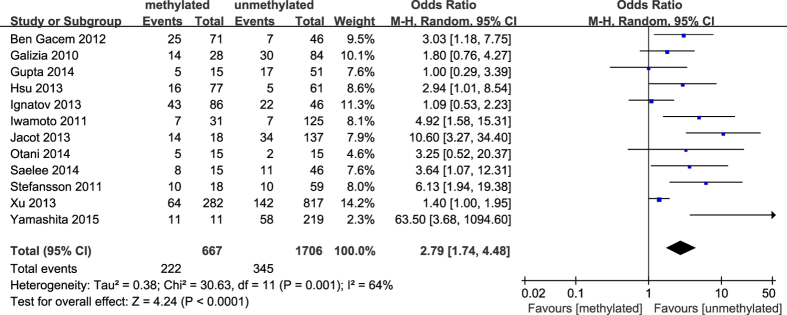
Forest plot for evaluating the association between BRCA1 promoter methylation and triple-negative phenotype. Random-effect model was used for the analysis.

**Figure 11:**
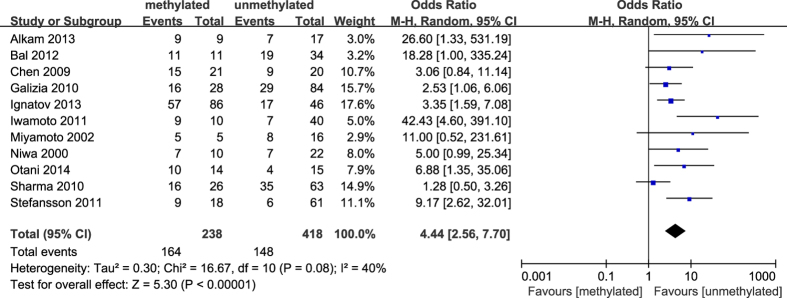
Forest plot for evaluating the association between BRCA1 promoter methylation and decreased or lack levels of BRCA1 protein expression. Random-effect model was used for the analysis.

The results of our meta-analysis showed that the *BRCA1* promoter methylation was statistically significant correlated with lymph node metastasis (OR = 1.25, 95%CI 1.06–1.48, P = 0.009, Fig. 3) and histological grade 3 (OR = 2.29, 95%CI 1.65–3.18, P < 0.001, Fig. 4), but had no correlation with early age (<50 years) at diagnosis (OR = 1.21, 95%CI 0.98–1.50, P = 0.07, Fig. 5) or premenopausal status (OR = 1.21, 95%CI 0.98–1.50, P = 0.08, Fig. 6). As for hormone receptors, strong associations of *BRCA1* methylation were found with ER negative (OR = 2.36, 95%CI 1.67–3.33, P < 0.001, Fig. 7) and also with PR negative (OR = 2.14, 95%CI 1.49–3.07, P < 0.001, Fig. 8). In contrast, no association was found between *BRCA1* methylation and Her2 status (OR = 1.58, 95%CI 0.98–2.56, P = 0.06, Fig. 9). Interestingly, our study confirmed that the *BRCA1* promoter methylation was positively correlated with triple-negative phenotype (OR = 2.79, 95%CI 1.74–4.48, P < 0.001, Fig. 10). Furthermore, it’s worth mentioning that there was statistically significant correlation between *BRCA1* methylation and decreased or lack expression levels of *BRCA1* protein (OR = 4.44, 95%CI 2.56–7.70, P < 0.001, Fig. 11). The detailed results were summarized in Table 4.

### Sensitivity analyses and publication bias

**Figure 12:**
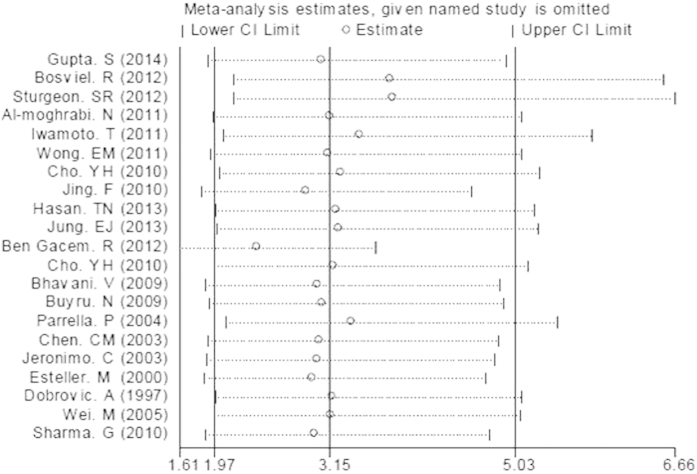
The plot of sensitivity analysis for evaluating the association between BRCA1 promoter methylation and breast cancer risk.

**Figure 13:**
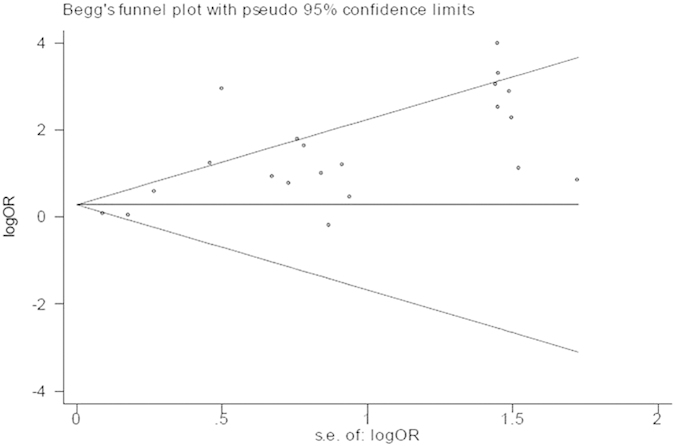
The funnel plot for evaluating the association of BRCA1 promoter methylation with breast cancer risk.

We performed sensitivity analyses to assess the robustness of the meta–analysis results by omitting each study in turn and no single study could essentially change the results. And the sensitivity analyses demonstrated that the results of our meta-analysis were statistically stable. Figure 12 showed the plot of sensitivity analysis for evaluating the association between *BRCA1* promoter methylation and breast cancer risk. The shapes of funnel plots and Egger’s linear regression test were used to evaluate the publication bias of the eligible literatures. In general, the funnel plots were not entirely symmetrical and the P-value of Egger’s test was not always greater than 0.05, indicating there was publication bias in our study. The funnel plot for evaluating the association of *BRCA1* promoter methylation with breast cancer risk was shown in Fig. 13 and the detailed results for P-value of Egger’s test were summarily in Table 3 and Table 4.

## Discussion

As is well-known that breast carcinoma is a heterogeneous group of tumors. It’s essential to find a reliable biomarker for the early diagnosis and prognosis prediction of breast cancer. In the meta-analysis, the strength of relationships of *BRCA1* promoter methylation with breast cancer risk and its clinicopathological features were systematically investigated. The results of our study confirmed that *BRCA1* promoter methylation was significantly correlated with the increased risk of breast cancer and associated with lymph node metastasis, histological grade 3, ER(-), PR(-), triple-negative phenotype and *BRCA1* protein expression, which indicated that *BRCA1* promoter methylation may be utilized as an effective biomarker in the management of breast tumors.

Our meta-analysis demonstrated that the prevalence of *BRCA1* promoter methylation in the breast cancer group was statistically significant elevated in comparison with the control group. This suggested a possibility that aberrant methylation of *BRCA1* promoter was correlated with an increased risk of breast cancer. And this almost was in line with the report by Wong *et al.* which confirmed that *BRCA1* methylation in peripheral blood DNA was correlated with 3.5-fold evaluated risk of having early-onset breast tumors^31^. In the stratification analysis based on sample materials, the summary OR was 4.75 in tissues and 1.87 in peripheral bloods, indicating that the association of *BRCA1* methylation with breast cancer risk in tissues was stronger than in peripheral bloods. Because there was only one study^26^ in which the tissue of the control group was derived from patients with benign breast diseases, we omitted the study to evaluate the frequency of *BRCA1* promoter methylation in breast cancer tissues compared with normal breast tissues, and the pooled OR was 7.23 (95%CI 4.35–12.01, P < 0.00001), which showed that the frequency of *BRCA*1 methylation in breast carcinoma tissues was 7.23-fold higher than that in normal breast tissues. The result demonstrated that the difference for the frequency of *BRCA1* methylation between breast tumors and non-cancerous breast tissues group was smaller than that between cancers and normal breast tissues group. Therefore, it suggested that normal breast tissues had a lower prevalence of *BRCA1* methylation than benign and malignant breast tissues, which also implied that the methylation of *BRCA1* gene promoter may play a certain role in the initiation of breast carcinoma. Similarly, a recent research confirmed that the *BRCA1* promoter methylation of histological normal breast epithelial cells may result in *BRCA1*-methylated breast tumors^47^. Additionally, our study demonstrated that the methylation of *BRCA1* promoter in peripheral blood DNA was correlated with a 1.87-fold increased risk of breast cancer, which was accordance with the result of a previous study^16^. The way of extracting DNA from peripheral blood is simple and barely invasive for detecting the methylation of *BRCA1* promoter. Therefore, the finding could open a new avenue for screening the risk of breast cancer.

In the subgroup analysis based on ethnicity, there were 15 studies in Caucasians, 4 in Asians and 2 in Australians for the association of *BRCA1* methylation with breast cancer risk. Meanwhile, significant difference in the level of *BRCA1* methylation was found in Caucasians (OR = 3.16), Asians (OR = 4.03) and Australians (OR = 3.27) in the cancer group compared with the control group, which suggested that different ethnicity and environment had certain impact on the prevalence of *BRCA1* methylation. Additionally, the results of stratified analysis based on methods showed that the different methods had different efficiency for detecting methylation. It’s essential to find an appropriate method based on feasibility of clinical practice in order to apply *BRCA1* methylation as a biomarker in clinical management.

In our meta-analysis, 18 articles discussed the association of *BRCA1* methylation with age at diagnosis in breast cancers. However, it’s meaningless to extract data from these articles to calculate the pooled OR and 95%CI on account of the difference in the definition of early age among these studies. Nevertheless, due to 10 articles considering early age as less than 50 years, we then investigated the correlation between *BRCA1* methylation and early age (<50 years) at diagnosis. However, there was no statistically significant association. In addition, no association between *BRCA1* methylation and premenopausal status was observed in our study. This was inconsistent with a previous report^43^ which showed that *BRCA1*-methylated breast cancers tended to occur at an early age (<50 years) and were more frequently observed in premenopausal or perimenopausal women than postmenopausal women. Furthermore, our results showed that *BRCA1* promoter methylation was strongly related to breast cancer patients with high histological grade and lymph node metastasis, revealing that aberrant methylation of *BRCA1* promoter may be implicated in the invasion and metastasis of breast cancer. In this sense, a report investigated the methylation profiles of 12 genes in the matched axillary lymph nodes compared with primary tumor tissues and the adjacent normal tissues from the same breast cancer patients, and demonstrated that the proportion of *BRCA1* methylation was higher in the matched axillary lymph nodes metastasis than normal tissue^50^. Thus, a hypothesis may be proposed that the methylation status of *BRCA1* promoter may be served as a biomarker for screening metastasis in breast tumors.

As expected from previous studies^14^,^29^,^43^, we demonstrated that there was a remarkable correlation between *BRCA1* promoter hypermethylation and breast tumors with lack of ER and PR expression. However, no inverse relationship was found between *BRCA1* methylation and Her2 status. Interestingly, it’s noteworthy that the triple negative phenotype was more common in *BRCA1*-methylated breast cancers than unmethylated tumors, which also was in agreement with numerous reports^18^,^32^,^44^,^45^. Altogether, these indicate that the patients with *BRCA1*-methylated breast tumors are likely to have little benefit from traditional hormone or targeted therapies. Additionally, a considerable amount of researches investigated that hypermethylation of *BRCA1* promoter resulted in the down-regulation of *BRCA1* expression^12^,^13^,^14^. Likewise, a statistically significant association of *BRCA1* methylation with lack or decreased expression of *BRCA1* protein was confirmed in our study. It’s interesting to note that there were positive and negative methylation-expression relationships in diverse gene regions, which differently affected genes expression and prognosis in breast cancer subtypes^51^. Therefore, it is reasonably predicted that methylation of BRCA1 different promoter region and distinct region of BRCA1 gene play different role in the BRCA1 expression and prognosis in breast tumors, and this needs further study.

Taken together, the clinicopathological features in sporadic *BRCA1*-methylated breast cancers compared with *BRCA1*-unmethylated tumors in our meta-analysis showed that sporadic breast carcinomas with *BRCA1* promoter methylation had molecular and clinicopathologic phenotype similar to those of hereditary BRCA1-mutated breast cancers, which was in line with several reports^27^,^37^. Furthermore, several lines of evidences confirmed that the expression pattern of sporadic *BRCA1*-methylated breast cancers was the same as that of inherited *BRCA1* mutations^52^. Herein, numerous preclinical researches investigated whether the antitumor activity of DNA-damaging agents in *BRCA1*-mutated breast cancers had a similar activity in *BRCA1*-methylated tumors, and the results demonstrated that the *BRCA1* hypermethylation conferred the same extent of sensitivity to poly adeno-sine diphosphate-ribose polymerase-1 (PARP1) inhibitors and platinum-derived drugs as did the BRCA1 mutation^53^,^54^,^55^. Moreover, Xu *et al.* reported that *BRCA1*-methylated triple-negative breast tumor patients were sensitive to adjuvant chemotherapy and had a significantly better 10-year disease-free survival (DFS) and disease-specific survival (DSS) than patients with *BRCA1*-unmethylated triple-negative tumors^46^. Importantly, a recent meta-analysis including 9 studies with 3205 breast cancer patients indicated that there was significant association of BRCA1 methylation with poor overall survival and DFS of breast tumors^56^. Hence, *BRCA1* promoter methylation may be a potential biomarker for targeted therapy and prognostic assessment.

Despite the advantage of a considerable number of included literatures, our meta-analysis had some limitations that should be thought over. First and most importantly, there was large heterogeneity in the outcome of the association between *BRCA1* promoter methylation and breast cancer risk. Nevertheless, stratification analyses based on sample materials, methods for detecting methylation or ethnicities showed to reduce the degree of heterogeneity among studies, which demonstrated that the three factors may be contributed to the heterogeneity. Additionally, there was large heterogeneity among studies for the correlation of *BRCA1* methylation with ER status, PR status and triple negative phenotype. The expression of ER and PR protein were almost assayed by immunohistochemical staining, but different antibody source and dilution ratio or even cut-off value for result evaluation should be taken into account for the source of heterogeneity. On the other hand, our included studies did not illustrate specific promoter regions of BRCA1 gene for methylation detection, and whether this may cause heterogeneity need to be further studied. Second, it’s noteworthy that there were no related articles for the prevalence of *BRCA1* promoter methylation in breast cancers in the African population among our eligible literatures. Therefore, further research is needed to evaluate whether this section of our results may be consistent with studies for the African ethnicity. Third, the control groups included healthy females and patients with benign breast diseases. No uniform definition of control groups may, to some extent, partly affected our research results. Finally, there was apparent publication bias in our study, which may be generated by defective design method of small sample studies or absent publication of small trials with negative results. In addition, language bias may be present on the basis of the fact that only English articles were included for our meta-analysis.

In conclusion, this meta-analysis indicated that *BRCA1* promoter methylation was associated with an increased risk of breast cancer. The prevalence of *BRCA1* methylation was, especially in mammary tissue, was high in patients with breast cancers compared with healthy females or patients with benign breast diseases. Furthermore, there were significant associations between *BRCA1* promoter methylation and clinicopathological characteristics in breast tumors, such as lymph node metastasis, high histological grade, ER-negative, PR-negative, triple-negative phenotype and reduced or lack expression of *BRCA1* protein. It’s necessary to need large-scale researches which use uniform criterion of control groups, detection methods for methylation and sample materials, before *BRCA1* promoter methylation can be a useful predictive or diagnostic biomarker for patients with breast cancer and applied to novel targeted therapeutic strategies in the future.

## Additional Information

**How to cite this article**: Zhang, L. and Long, X. Association of *BRCA1* promoter methylation with sporadic breast cancers: Evidence from 40 studies. *Sci. Rep.* **5**, 17869; doi: 10.1038/srep17869 (2015).

## Figures and Tables

**Table 1:** Characteristics of studies included for the association between BRCA1 promoter methylation and breast cancer risk in the meta-analysis.

First author	Year	Country	Case		Control		Methods	Materials	Control characteristics
			M+	M−	M+	M−			
Gupta. S^33^	2014	Poland	15	51	2	34	MS-HRM	Blood	Female without breast cancers
Bosviel. R^5^	2012	France	425	477	454	536	QAMA	Blood	Female without breast cancers
Sturgeon. SR^3^	2012	USA	192	112	147	87	Pyrosequencing	Blood	Benign breast disease controls
Al-moghrabi. N^30^	2011	Saudi Arabic	2	5	8	65	MSP	Blood	Healthy female
Iwamoto. T^16^	2011	Japan	43	157	27	173	QMSP	Blood	Female without breast cancers
Wong. EM^31^	2011	Australia	28	227	6	163	MS-HRM	Blood	Female without breast cancers
Cho. YH^17^	2010	USA	3	37	2	38	MethyLight	Blood	Ethnicity-matched healthy female
Cho. YH^17^	2010	USA	7	33	2	25	MethyLight	Tissue	Paired adjacent normal breast tissue
Jing. F^2^	2010	China	17	33	0	50	MSP	Blood	Female without breast cancers
Sharma. G^29^	2010	India	25	75	0	30	MSP	Blood	Healthy female
Hasan. TN^6^	2013	India	9	20	4	22	MSP	Tissue	Normal breast biopsies
Jung. EJ^4^	2013	Korea	6	54	3	57	MS-MLPA	Tissue	Paired adjacent normal breast tissue
Ben Gacem. R^32^	2012	Tunisia	71	46	5	60	MSP	Tissue	Paired adjacent normal breast tissue
Bhavani. V^28^	2009	India	21	83	2	46	MSP	Tissue	Paired adjacent normal breast tissue
Buyru. N^15^	2009	Turkey	4	73	0	77	MS-MLPA	Tissue	Paired adjacent normal breast tissue
Wei. M^27^	2005	USA	39	92	0	3	MSP	Tissue	Paired adjacent normal breast tissue
Parrella. P^26^	2004	Italy	9	45	2	8	MSP	Tissue	Benign breast disease
Chen. CM^24^	2003	China	21	72	0	20	MSP	Tissue	Paired adjacent normal breast tissue
Jerónimo C^25^	2003	Portugal	11	16	0	12	MSP	Tissue	Paired adjacent normal breast tissue
Esteller. M^10^	2000	USA	11	73	0	84	MSP	Tissue	Paired adjacent normal breast tissue
Dobrovic. A^23^	1997	Australia	2	5	0	2	Southern blotting	Tissue	Normal breast biopsies

M+: methylated; M−: unmethylated; MSP: methylation-specific PCR; MS-HRM: methylation-sensitive high-resolution melting; QMSP: methylation-specific quantitative PCR; QAMA: quantitative analysis of methylation alleles; MS-MLPA: methylation-specific multiplex ligation-dependent probe amplification.

**Table 2:** Characteristics of studies included for the association between BRCA1 promoter methylation and clinicopathologic features of breast cancer in the meta-analysis.

First author	Year	Country	Number of patients	Method	Materials	Patients characteristics
Zhu. X^49^	2015	China	239	MSP	Tissue	sporadic primary TNBC
Yamashita. N^48^	2015	Japan	230	COBRA	Tissue	TNBC and non-TNBC
Saelee. P^42^	2014	Thailand	61	MSP	Tissue	invasive ductal breast cancer
Gupta. S^33^	2014	Poland	66	MS-HRM	Blood	TNBC or medullary breast cancer
Otani. Y^47^	2014	Japan	30	QMSP	Tissue	Female primary breast cancer patients
Hasan. TN^6^	2013	India	29	MSP	Tissue	sporadic breast cancer
Jung. EJ^4^	2013	Korea	60	MS-MLPA	Tissue	primary breast tumors
Alkam. Y^12^	2013	Japan	26	MSP	Tissue	Basal-like breast cancer
Hsu. NC^18^	2013	China	139	MSP	Tissue	early-stage sporadic breast cancer
Jacot. W^45^	2013	France	155	MSP	Tissue	sporadic breast cancer
Ignatov. T^13^	2013	Germany	132	MSP	Tissue	sporadic TNBC
Xu. Y^46^	2013	China	1163	MSP	Tissue	operable primary breast cancer
Ben Gacem. R^32^	2012	Tunisia	117	MSP	Tissue	sporadic breast cancer
Sturgeon. SR^3^	2012	USA	304	Pyrosequence	Blood	operable breast cancer
Bal. A^14^	2012	India	45	MSP	Tissue	sporadic breast cancer
Iwamoto. T^16^	2011	Japan	162	QMSP	Tissue	operable breast cancer
Singh. AK^43^	2011	India	127	MSP	Tissue	sporadic breast cancer
Stefansson. OA^44^	2011	Iceland	79	MSP	Tissue	sporadic breast cancer
Al-moghrabi. N^30^	2011	Saudi Arabia	46	MSP	Tissue	sporadic breast cancer
Wong. EM^31^	2011	Australia	236	MS-HRM	Blood	breast cancer before the age of 40 years
Galizia. E^40^	2010	Italy	112	MSP	Tissue	sporadic TNBC
Sharma. G^29^	2010	India	89	MSP	Tissue	operable primary breast cancer
Xu. X^41^	2010	USA	851	MSP	Tissue	primary invasive or *in situ* breast cancer
Chen. Y^39^	2009	China	41	MSP	Tissue	sporadic breast cancer
Jing. F^38^	2008	China	102	MSP	Blood	sporadic breast cancer
Birgisdottir. V^37^	2006	Iceland	143	MSP	Tissue	sporadic breast cancer
Wei. M^27^	2005	USA	125	MSP	Tissue	sporadic breast cancer
Miyamoto. K^36^	2002	Japan	21	bisulfite sequence	Tissue	sporadic breast cancer
Niwa. Y^35^	2000	Japan	32	MSP	Tissue	sporadic breast cancer
Catteau. A^34^	1999	UK	96	southern	Tissue	sporadic breast cancer

MSP: methylation-specific; MS-HRM: methylation-sensitive high-resolution melting; QMSP: methylation-specific quantitative PCR; MS-MLPA: methylation-specific multiplex ligation-dependent probe amplification; COBRA: combined bisulfite and restriction analysis;TNBC: triple-negative breast cancer.

**Table 3:** Stratified analysis of the frequency of BRCA1 promoter methylation in breast cancers compared with non-cancer controls.

Studies	N	OR	95%CI	P	P_H_	I^2^	P_bias_
Total	21	3.15	1.97–5.03	<0.001	<0.001	74%	0.0001
Materials							
Tissue	12	4.75	2.37–9.54	<0.001	0.09	38%	0.776
Blood	9	1.87	1.19–2.96	0.007	0.001	69%	0.002
Methods							
MSP	11	6.79	3.05–15.11	<0.001	0.05	45%	0.825
Others	10	1.53	1.09–2.14	0.01	0.05	46%	0.005
Ethnic							
Caucasians	15	3.16	1.78–5.62	<0.001	<0.001	77%	0.002
Asians	4	4.03	1.07–15.18	0.04	0.05	63%	0.119
Australians	2	3.27	1.37–7.84	0.008	0.83	0%	–

N: the total number of eligible studies; P_H_: the p-value of Q test for heterogeneity among studies. P_bias_: the p-value of Egger linear regression test for evaluating publication bias.

**Table 4:** The association between clinicopathological features and BRCA1 promoter methylation compared with BRCA1-unmethylated breast cancer.

Clinicopathological characteristics	N	Methylation		Heterogeneity		Publication
		OR (95%CI)	P	I^2^	P	P_bias_
lymph node metastasis	17	1.25 (1.06–1.48)	0.009	7%	0.37	0.273
histological grade 3	18	2.29 (1.65–3.18)	<0.001	41%	0.04	<0.001
early age (<50 years)	10	1.21 (0.98–1.50)	0.07	6%	0.38	0.311
premenopausal status	11	1.21 (0.98–1.50)	0.08	5%	0.39	0.506
ER (-)	18	2.36 (1.67–3.33)	<0.001	60%	0.0006	<0.001
PR (-)	16	2.14 (1.49–3.07)	<0.001	61%	0.0007	<0.001
Her2 (-)	10	1.58 (0.98–2.56)	0.06	45%	0.06	0.007
TNBC	12	2.79 (1.74–4.48)	<0.001	64%	0.001	0.007
BRCA1 expression (-)	11	4.44 (2.56–7.70)	<0.001	40%	0.08	0.009

N: the total number of eligible studies.

P_bias_: the p-value of Egger linear regression test for evaluating publication bias.
